# *Corynebacterium glutamicum*, a natural overproducer of succinic acid?

**DOI:** 10.1002/elsc.201900141

**Published:** 2020-01-20

**Authors:** Amani Briki, Karim Kaboré, Eric Olmos, Sabine Bosselaar, Fabrice Blanchard, Michel Fick, Emmanuel Guedon, Frantz Fournier, Stéphane Delaunay

**Affiliations:** ^1^ Laboratoire Réactions et Génie des Procédés CNRS Vandoeuvre Cedex France; ^2^ Laboratoire Réactions et Génie des Procédés Université de Lorraine Vandoeuvre Cedex France

**Keywords:** *Corynebacterium glutamicum*, oxygen transfer, residual glucose concentration, succinate

## Abstract

*Corynebacterium glutamicum* is well known as an important industrial amino acid producer. For a few years, its ability to produce organic acids, under micro‐aerobic or anaerobic conditions was demonstrated. This study is focused on the identification of the culture parameters influencing the organic acids production and, in particular, the succinate production, by this bacterium. *Corynebacterium glutamicum* 2262, used throughout this study, was a wild‐type strain, which was not genetically designed for the production of succinate. The oxygenation level and the residual glucose concentration appeared as two critical parameters for the organic acids production. The maximal succinate concentration (4.9 g L^−1^) corresponded to the lower *k_L_a* value of 5 h^−1^. Above 5 h^−1^, a transient accumulation of the succinate was observed. Interestingly, the stop in the succinate production was concomitant with a lower threshold glucose concentration of 9 g L^−1^. Taking into account this threshold, a fed‐batch culture was performed to optimize the succinate production with *C. glutamicum* 2262. The results showed that this wild‐type strain was able to produce 93.6 g L^−1^ of succinate, which is one of the highest concentration reported in the literature.

**Abbreviations**
DOC: dissolved oxygen concentration
*k_L_a*: volumetric mass transfer coefficient
LDH: lactate dehydrogenase

## 1 INTRODUCTION

*Corynebacterium glutamicum* has become a platform organism for white biotechnology widely used in industry for the production of amino acids such as lysine and glutamate 1, 2. Several studies have demonstrated its ability to produce a variety of other metabolites, such as organic acids in oxygen‐deprived conditions 3, 4, 5. The impact of such culture conditions on the growth of *C. glutamicum* remains discussed. In this environment, *C. glutamicum* R produced lactate, succinate, and acetate without significant growth 6. It was also previously assumed that *C. glutamicum* ATCC13032 can grow anaerobically only in the presence of nitrate 7, 8. However, recently, it was demonstrated that, even in the absence of nitrate, this same strain exhibited a residual growth, in anaerobic conditions, using glucose, fructose, sucrose, or ribose as substrates and with lactate, succinate, and acetate as products 9.

The previously mentioned organic acids are the main products identified during culture of *C. glutamicum* under oxygen‐deprived conditions. One of them, succinate, classified by U.S. Department of Energy as one of the top 12 building block chemicals from biomass, has a wide variety of applications such as surfactant, foaming agent, food, or pharmaceutical additive. It can also serve also a platform for the synthesis of other chemicals as 1.4‐butanediol and tetrahydrofuran, which can be used in polymer synthesis 10, 11. Metabolic and/or process engineering strategies have been applied to improve the succinate production in *C. glutamicum*. However, it is accepted in the scientific literature that the optimization of the succinate production by *C. glutamicum* necessitates a genetic improvement of this bacterium. In anaerobic conditions, succinate is synthesized via glycolysis by carboxylation of PEP or pyruvate to oxaloacetate and through the reductive branch of the TCA cycle 6. The addition of CO_2_, in gaseous form or as carbonate salt, enhanced the production rate and yield of succinate produced by wild‐type or metabolically engineered strains of *C. glutamicum* 4, 6, 12. Okino et al. 4 developed a two‐step process for succinate production in which cells grew aerobically, were harvested, washed, and transferred in a second bioreactor with anaerobic conditions for the organic acid production. Using this two‐step process, an engineered strain was able to produce 146 g L^−1^ succinate with a yield of 1.4 mol mol^−1^ glucose, after 46 h of culture 13. With this strain, no lactate production was observed, but a large amount of acetate was produced. Later, Litsanov et al. 14 engineered *C. glutamicum* ATCC13032 to reduce the acetate production. The resultant strain was able to produce 134 g L^−1^ succinate with a yield of 1.67 mol mol^−1^ glucose using the two‐step process with an anaerobic fed batch phase. Alternatively, an one‐step process with successive aerobic transition and anaerobic phases in the same bioreactor was set up 15. These last authors demonstrated that the transition from aerobic to anaerobic conditions, defined as micro‐aerobic conditions, had a strong influence on organic acid production. According to Kaboré et al. 15, micro‐aerobic conditions consist in a constantly limiting oxygen transfer rate but with non‐zero dissolved oxygen concentrations. Similarly, Byun et al. 16 characterized this oxygenation state as a phase with low dissolved oxygen concentration (0–5%). It was also shown, by determination of the volumetric mass transfer coefficient (*k_L_a*) during cultures in shake flask and using a mutant strain of *C. glutamicum* deleted for lactate dehydrogenase (LDH), that a critical oxygenation level had to be defined according to the produced organic acid. The optimal transfer condition corresponded to *k_L_a* values between 10 and 19 h^−1^ for succinate production and, 19 and 33 h^−1^ for acetate production 15. Additionally, different strategies of transition from the aerobic to anaerobic conditions have been studied. Compared to an instantaneous transition from aerobiosis to anaerobiosis, applying an optimized transition strategy led to an increase in the succinate concentration up to 640% 15. This positive impact of a micro‐aerobic environment on the synthesis of succinate was also observed in *E. coli*. The introduction of a micro‐aerobic phase at the end of the aerobic growth phase resulted in a significant increase in the expression of genes involved in the succinate synthesis pathway, and thus improved the succinate yield and productivity during the anaerobic phase 17.

**PRACTICAL APPLICATION**

Succinate is one of the 12 top bio‐based building blocks. Several strategies have been developed to optimize the bioproduction of this di‐acid such as identification of natural overproducing microbial strains or genetic design of current industrial microorganisms, mainly *Escherichia coli* or *Corynebacterium glutamicum*. Nevertheless, the understanding of the impacts of the culture parameters on the physiology of the microorganisms may also constitute an accurate approach to improve the production of organic acids by non‐genetically designed microorganisms. Then, based on the understanding of the physiological behaviour of *C. glutamicum*, a GRAS bacterium, a process with a controlled oxygen transfer rate was proposed for the production of organic acids including succinic acid. Applying this process allowed the production of one of the highest concentration of succinate reported to date with a non‐genetically modified microorganism.

The capacity of the microbial biocatalysts to re‐consume the product of interest may affect the performance of some bioprocesses. Concerning the production of succinate by *C. glumaticum*, a decrease in the succinate concentration, in micro‐aerobic environments, was previously noticed at the end of cultures using a strain deleted for LDH. This re‐consumption was related, according to the authors, to the glucose depletion in the culture medium 18. Moreover, recently, Conrady et al. 19 showed that *C. glutamicum* was able to simultaneously consume glucose and the produced organic acids (lactate, acetate, and succinate) when the oxygenation of the culture medium was sufficient. These works suggest that the understanding and the control of the succinate consumption by *C. glutamicum* may contribute to enhance the performances of succinate production by this bacterium.

Therefore, this study aimed to evaluate the physiological behaviour of a wild‐type strain of *C. glutamicum* in an oxygen‐deprived environment and to compare it with those strains designed for the succinate production. The second goal of the study was to investigate the ability of *C. glutamicum* to consume the previously produced succinate. Then, based on the collected data and the data from the literature, and using only a process engineering strategy, the potential of *C. glutamicum* in terms of succinate production was determined.

## 2 MATERIALS AND METHODS

### 2.1 Strain and culture medium

*Corynebacterium glutamicum* 2262 was used in this study. This non‐genetically engineered strain, initially used for the glutamate overproduction 20 was cultivated in a modified MCGC mineral salt medium in which the citrate was replaced by deferoxamine. The medium compositions for the culture in shake flask and in bioreactor can be found in Supporting Information Table S1.

### 2.2 Determination of the volumetric mass transfer coefficient *k_L_a*

The *k_L_a* (h^−1^) can be determined by experimental method or by empirical model. In our study, we used the empirical correlation of Seletzky et al. 21 based on the operating parameters as the shaking frequency (*N*, rpm), filling volume (*V*, mL), the maximal shake flask diameter (*d*, cm), and shaking diameter (*d_0_*, cm; Equation (1)). This model is based on data obtained from a chemical model system (sodium sulfite) in which the catalyst, cobalt sulfate, simulated a microbial oxygen consumption. It was successfully used with several microbial catalysts such as *C. glutamicum* ATCC13032 21 and *Streptomyces pristinaespiralis* 22. Moreover, using a *ldhA*‐deleted strain of *C. glutamicum* 2262, Kaboré et al. 18 demonstrated the very good agreement between experimental and estimated values with a relative difference less than 10%.

(1) $$k_{L}a=0.024\times N^{1.16}\times V^{-0.83}\times d_{0}^{0.38}\times d^{1.92}$$

This correlation was validated for a broad range of the operating parameters (*N*: 50–500 rpm; *V*: 4–20%; *d_0_*: 1.25‐10 cm, and flask volumes between 50 and 1000 mL), and the accuracy was 30% 21.

### 2.3 Culture conditions

A first preculture (A) consisted in 50 mL of MCGC, contained in a 500 mL baffled shake flask, inoculated with 1 mL of a glycerol stock of *C. glutamicum* 2262. The preculture (A) was performed at a temperature of 33°C and an agitation rate of 200 rpm, until reaching an optical density at 570 nm of 60. Five milliliters of this preculture (A) were used to inoculate a second preculture (B) performed in the same operating conditions. When the optical density at 570 nm reached the value of 30, the preculture (B) was stopped by immersing the flask in 70% v/v ethanol, at a temperature of −20°C. Then, the flask was stored at a temperature of 4°C for 24 h maximum. Preculture (B) was used as inoculum for the culture experiments in flasks. All the cultures were performed in unbaffled shake flasks. By varying the shaking frequency (*N*), the flask maximal volume (*V_T_*), the filling volume (*V_L_*), the flask diameter (*d*) and the shaking diameter (*d_0_*), different *k_L_a*, and then, oxygenation conditions were applied. All the culture conditions are listed in Supporting Information Table S2. To avoid modification in the oxygenation conditions by the volume changes due to samplings, for each *k_L_a* condition, several flasks were used in parallel. Each flask was sacrificed for the determination of biomass and organic acid concentrations at a single culture time and in a specific *k_L_a* condition. For a *k_L_a* about 90 h^−1^, other cultures were performed by adding succinate and or glucose in the medium. Succinate was dissolved in water, adjusted to pH 7.6 with KOH, and sterilized by filtration. All cultures were performed at 33°C and the initial pH was adjusted to 7.6 with KOH.

### 2.4 One‐step process for the organic acid production using *C. glutamicum* 2262

The culture was performed in a 2 L stirred tank bioreactor equipped with pH, O_2_, and temperature probes. During the culture, the temperature was maintained at a value of 33°C. The pH was regulated at a set point of 7.6 by automatic addition of a 10 N KOH solution or 2 N HCl solution using a peristaltic pump. The agitation rate was maintained at a value of 900 rpm. The initial culture medium was 1.13 L of MCGC medium containing 85.5 g L^−1^ of glucose combined with 14 g L^−1^ of NaHCO_3_. A feeding was applied after the transition phase, once the aeration was totally stopped. Eight pulses of 50 mL of glucose at a concentration of 630 g L^−1^ and 50 mL of NaHCO_3_ solution at a concentration of 84 g L^−1^ were performed after 10, 18, 24, 28, 42, 48, 53, and 68 h, respectively.

A one‐step process based on three successive aerobic, micro‐aerobic, and anaerobic phases was used for the succinate production by *C. glutamicum* 2262. During the aerobic phase, the dissolved oxygen concentration (DOC) was maintained at a constant concentration of 50% of air saturation by changing the airflow rate. When the cells reached the optical density of about 30 at 570 nm, a progressive deoxygenation based on the control of dissolved oxygen concentration was applied. In this study, the best transition strategy proposed by Kaboré et al. 15 was applied: the dissolved oxygen level was switched from 50 to 20% then to 10% of air saturation. The DOC was maintained for at least 2 h for each level. At the end of the transition phase, the aeration was totally stopped that initiated the anaerobic phase.

### 2.5 Analytical methods

The biomass concentration was determined by the measurement of absorbance at 570 nm with a Thermos Scientific Multiskan GO (Thermo Fisher Scientific, France). The dry cell concentration was calculated using the following relation:

(2) $$\left(g\;\mathrm{DW}\;L^{-1}\right)=0.35\times OD_{570\;\mathrm{nm}}$$

The lactate, succinate, acetate, pyruvate, and fumarate concentrations were measured by HPLC according to the method described by Kaboré et al. 18.

### 2.6 Determination of kinetic parameters

The global kinetic parameters were determined for each experiment. Maximal specific growth, glucose consumption, and organic acid production rates were calculated as follows:

$$\;\mu_{max}=\;max_{t}\left(\frac{1}{{\left[X\right]}_{f}}\;\times \frac{d{\left[X\right]}_{f}}{dt}\right)$$

$$\;q_{max}^{OA}=\;max_{t}\left(\frac{1}{{\left[X\right]}_{f}}\;\times \frac{d{\left[OA\right]}_{f}}{dt}\right)$$

$$\;q_{max}^{glu}=\;-max_{t}\left(\frac{1}{{\left[X\right]}_{f}}\;\times \frac{d{\left[glu\right]}_{f}}{dt}\right)$$

where [*X*]_f_, [*OA*]_f_, and [*glu*]_f_ were fitted concentrations of biomass, organic acids, and glucose, respectively. The fitting was performed using smoothing spline interpolation method (Matlab, Mathworks, USA).

## 3 RESULTS

### 3.1 Effect of gas–liquid mass transfer on the growth, the glucose consumption, and the organic acids production

**Figure 1:**
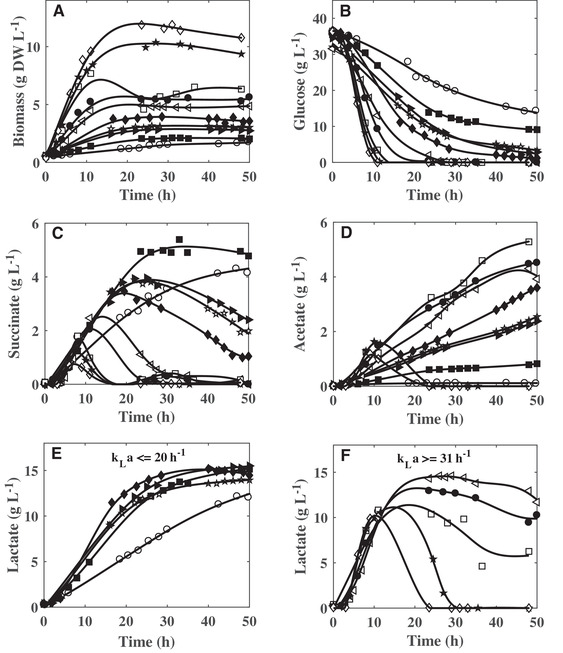
Influence of volumetric mass transfer coefficient (*k_L_a*) values on the (A) biomass, (B) glucose, (C) succinate, (D) acetate, and (E and F) lactate production by *C. glutamicum* 2262. The values of *k_L_a* were 0.6 (○), 5 (■), 11 (☆), 15 (▲), 20 (♦), 31 (⊲), 44 (•), 77 (□), 90 (★), and 118 h^−1^ (◊)

To investigate the impact of oxygenation level on the physiological response of the wild‐type strain, *C. glutamicum* 2262, batch cultures were performed in unbaffled shake flasks. As it was previously validated for a mutant strain of *C. glutamicum* 2262 18, the correlation of Seletzky et al. 21 was used for the evaluation of applied *k_L_a* during the cultures. This parameter was set at 0.6, 5, 11, 15, 20, 31, 44, 77, 90, and 118 h^−1^, respectively. Time profiles of biomass, glucose, and organic acid concentration collected during these different experiments are presented in Figure 1. Pyruvate and fumarate were also detected, in addition of lactate, succinate, and acetate. However, fumarate and pyruvate concentrations remained below 0.12 and 0.44 g L^−1^, respectively, and were not significantly influenced by the changes in the gas–liquid mass transfer (data not shown).

**Figure 2:**
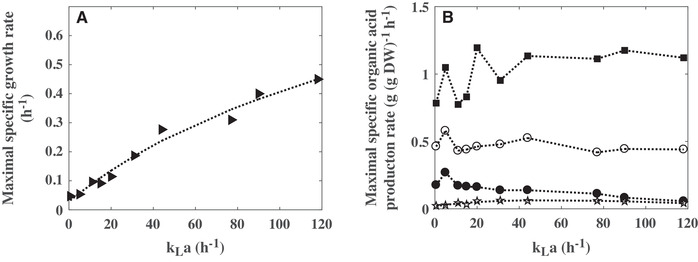
Influence of the volumetric mass transfer coefficient (*k_L_a*) on (A) the maximal specific growth rate, (B) the maximal specific glucose consumption rate (■), and the maximal specific lactate (**○**), succinate (•), and acetate (☆) production rate by *C. glutamicum* 2262

Increasing the *k_L_a* value from 0.6 to 118 h^−1^ led to an increase of the maximal concentration of biomass from 2 to 12 g L^−1^ (Figure 1A). Up to 44 h^−1^, the *k_L_a* had also a strong influence on the maximal specific growth rate. Above this threshold, the bacterial growth was less impacted (Figure 2A).

In this study, glucose was used as the sole initial carbon source. Increasing the gas–liquid mass transfer induced an earlier depletion of glucose (Figure 1B). While the glucose was not entirely consumed during the cultures with *k_L_a* under 20 h^−1^, glucose exhaustion was observed from *k_L_a*  =  31 h^−1^ and higher. Residual concentrations of 1.3, 2.9, 3.1, 9, and 13.1 g L^−1^ glucose were determined in the supernatant of the cultures at *k_L_a* values of 20, 15, 11, 5, and 0.6 h^−1^, respectively. The glucose was consumed with the same maximal specific consumption rate, about 1.15 g (g DW)^−1^ h^−1^, for *k_L_a* values higher than 20 h^−1^ (Figure 2B).

**Figure 3:**
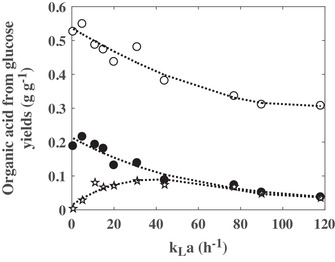
Influence of the volumetric mass transfer coefficient (*k_L_a*) on the yields of lactate (**○**), succinate (•), and acetate (☆), from glucose

In all the culture conditions, the production of lactate, succinate, and acetate was noticed (Figure 1C–F). The lactate appeared always as the main product. Remarkably, the maximal lactate concentration remained very similar for *k_L_a* values between 5 and 31 h^−1^, with concentrations between 14.4 and 15.3 g L^−1^ (Figure 1E), and decreased for higher *k_L_a* values (Figure 1F). Above 31 h^−1^, the accumulation of lactate was transitory, which is due to the consumption of the previously produced lactate. This use of lactate as a substrate was closely correlated to the glucose exhaustion in the culture medium (Figure 1B,F). The lactate from glucose yield decreased with the increase in the *k_L_a* value. The highest lactate yields, about 0.55 g g^−1^ glucose, were reached for *k_L_a* values of 0.6 and 5 h^−1^ (Figure 3). Similarly, the optimal specific production rate of lactate was obtained at *k_L_a* value of 5 h^−1^ (Figure 2B).

The second organic acid, in a quantitative point of view, was acetate. Up to 5.3 g L^−1^ were accumulated at 77 h^−1^. The synthesis of this organic acid was drastically impacted by the *k_L_a*. Its maximal concentration increased with the *k_L_a* from 0.6 to 77 h^−1^. Then, it was only transiently produced at 90 and 118 h^−1^ (Figure 1D). The acetate reassimilation was concomitant with the lactate reconsumption (Figure 1D,F). As the maximal concentration, the acetate from glucose yield and the specific acetate production rate displayed a maximum value according to the gas‐liquid mass transfer coefficient. The maximum yield was measured for *k_L_a* ranging from 31 to 77 h^−1^ (Figure 3), whereas the maximal specific acetate production rate reached a plateau, with maximum value close to 0.06 g (g DW)^−1^ h^−1^, for *k_L_a* values between 20 and 90 h^−1^ (Figure 2B).

**Figure 4:**
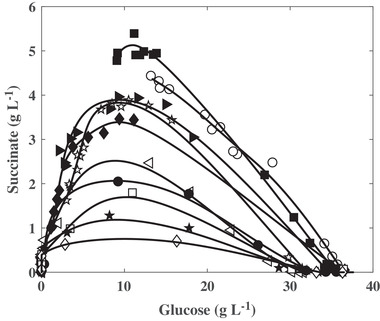
Dependence of succinate production by *C. glutamicum* 2262, on the residual glucose concentration for the different *k_L_a* conditions. The values of *k_L_a* were 0.6 (○), 5 (■), 11 (☆), 15 (*►*), 20 (♦), 31 (⊲), 44 (•), 77 (□), 90 (★), and 118 h^−1^ (◊)

Additionally, *C. glutamicum* 2262 produced succinate during the different cultures. The maximal succinate concentration (4.9 g L^−1^) and conversion yield (0.22 g g^−1^ glucose) corresponded to lowest *k_L_a* values, 0.6 and 5 h^−1^ (Figures 1C and 3). Similarly, the highest maximal specific succinate production rate was obtained to the *k_L_a* value of 5 h^−1^. A decrease in the succinate maximal concentration and the maximal specific succinate production rate was then observed when increasing the *k_L_a* value. Interestingly, except at 0.6 and 5 h^−1^, the succinate accumulation in the culture broth was transient. The stop of the net succinate production occurred earlier during the culture as the *k_L_a* increased, leading to a lower succinate final concentration for *k_L_a* between 11 and 20 h^−1^ and to close to zero at the end of the cultures for *k_L_a* above 31 h^−1^. It is noticeable that the succinate reassimilation started while glucose was still available in the culture broth for bacterial growth and energetic needs. To better put that into evidence, the succinate concentrations were plotted versus the glucose residual concentrations. In all the gas–liquid mass transfer conditions, the succinate re‐assimilation occurred at a lower threshold glucose concentration of about 9 g L^−1^ (Figure 4). A concomitant consumption of both succinate and glucose was then measured until the complete depletion of the latter.

### 3.2 Succinate as a co‐substrate for *C. glutamicum* 2262

**Figure 5:**
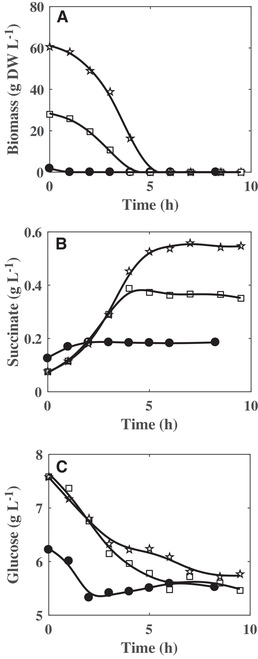
Kinetics of biomass concentration (A), succinate (B), and glucose (C) consumptions during cultures of *C. glutamicum* 2262 in unbaffled shake flask at a *k_L_a* value of 90 h^−1^ and with initial concentrations of 4.1 g L^−1^ succinate and no glucose (•), 4.9 g L^−1^ succinate plus 5 g L^−1^ glucose (□), and 4.9 g L^−1^ succinate plus 10.8 g L^−1^ glucose (☆)

In order to determine the role of succinate as a substrate, cultures were performed with succinate as sole carbon source or with a mixture of glucose and succinate (Figure 5). In all these cultures, a *k_L_a* value of 90 h^−1^ was imposed. The traces of glucose (0.3 g L^−1^) initially present in the culture in which succinate should be the sole carbon source were brought by the inoculum. In all the cultures, bacterial growth was noticed only before glucose exhaustion. This growth stopped after 1, 4, or 5 h in presence of 4.1 g L^−1^ succinate, 4.9 g L^−1^ succinate plus 5 g L^−1^ glucose, or 4.9 g L^−1^ succinate plus 10.8 g L^−1^ glucose, respectively. In all the conditions, succinate was consumed simultaneously to glucose. The succinate consumption ceased with the exhaustion of glucose when no glucose was initially added in the culture medium, whereas the succinate was still slowly metabolized until the end of the culture in the two other conditions. However, this organic acid was clearly not able to sustain the growth of *C. glutamicum* when it remained the only available carbon molecule in the culture medium (Figure 5A,B).

### 3.3 Succinate production by *C. glutamicum* 2262 in a one‐step process

**Figure 6:**
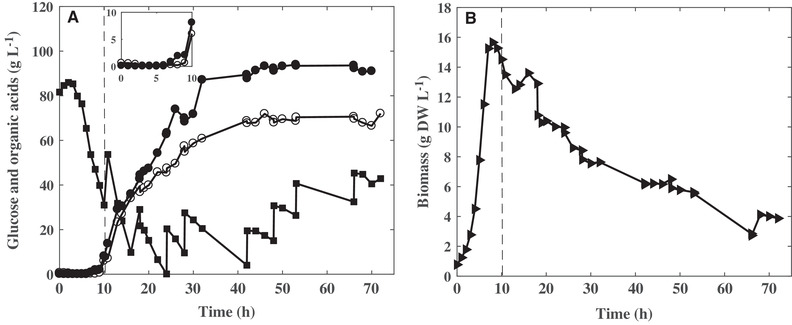
Fed‐batch culture of *C. glutamicum* 2262. Kinetics of (A) glucose (■), succinate (•), lactate (○) concentrations, and (B) biomass concentration. The dashed vertical line indicates the beginning of the anaerobic phase. The insert graph in part (A) presents the kinetics of organic acids corresponding to the transition phase (between 4 and 10 h culture)

As demonstrated above, the succinate synthesis in *C. glutamicum* is impacted by the oxygenation level and the residual glucose concentration. In addition to these two parameters, it was previously shown that the succinate production might be limited by the dissolved CO_2_ sustainability due to the crucial role of anaplerotic enzymes in the succinic acid biosynthetic pathway 6. Therefore, to evaluate the potential of *C. glutamicum* 2262 to produce the succinate, a fed‐batch culture was performed by applying the one‐step process with, successively, aerobic, optimized transition and anaerobic phases previously developed with a *ldhA* deleted strain of *C. glutamicum* 2262 15. The culture started with an initial volume of 1.2 L MCGC medium (containing 85.5 g L^−1^ glucose and 14.1 g L^−1^ NaHCO_3_). During the anaerobic phase, eight additions of 50 mL glucose (630 g L^−1^) and 50 mL NaHCO_3_ (84 g L^−1^) were performed. Under aerobic conditions (until 6 h of culture), glucose was metabolized to biomass and no production of organic acid was observed (Figure 6). During the transition phase (from 6 to 10 h of culture), *C. glutamicum* kept growing and reached a maximal mass of about 18 g DW (about 16 g DW L^−1^). Few amounts of organic acids were produced. At the end of this phase, the growth stopped whereas a residual glucose concentration of about 30.9 g L^−1^ was measured. At the beginning of the anaerobic phase, the first pulse of glucose and bicarbonate solution was done. A rapid increase in the organic acid production was observed while the biomass concentration decreased to reach a final concentration of 3.87 g DW L^−1^ (7.5 g DW). The glucose concentration remained higher than 9 g L^−1^ except at 24 and 42 h. Under these conditions, *C. glutamicum* 2262 produced mainly lactate and succinate with a succinate to lactate ratio of about 1 mole per mole. At the end of the culture, 1.1 g L^−1^ of pyruvate were produced and no acetate was detected (data not shown). After the fifth pulse, the organic acid production reached a plateau with a maximal concentration of succinate and lactate of about 93.6 g L^−1^ (793 mM) and 70.7 g L^−1^ (785 mM), respectively. The glucose was accumulated to 42.8 g L^−1^ at the end of the culture.

## 4 DISCUSSION

To date, the succinate production using *C. glutamicum* was mainly performed with genetically modified strains. It is generally assumed that the deletion of *ldhA* (lactate dehydrogenase encoding gene) is the minimal genetic modification to achieve the succinate overproduction with this bacterium. The absence of NADH cofactors re‐oxidation, consequently to the lack of lactate dehydrogenase, drives the carbon flux towards the succinate synthesis pathway in which the NADH re‐oxidation occurs at the malate dehydrogenase and succinate dehydrogenase levels. Up to now, only one publication used a wild‐type strain, *C. glutamicum* ATCC13032, to investigate the influence of CO_2_ availability on the succinate production with a focus on modification in the intracellular carbon fluxes induced by this CO_2_ supplementation 12. In the present study, the potential of a wild‐type strain of *C. glutamicum* in terms of succinate production was investigated.

Under deprived oxygen or anaerobic conditions, *C. glutamicum* metabolized glucose to produce organic acids, mainly lactate, succinate and acetate 4, 6. In this study, organic acid production was clearly shown to be related to the culture medium oxygenation (Figure 1). The final concentration of lactate, which is the main fermentation end‐product, was maximal when *k_L_a* value was set between 5 and 20 h^−1^. The optimal succinate production corresponded to *k_L_a* of 5 h^−1^ and below. For *k_L_a* values between 31 and 77 h^−1^, the production of lactate and succinate decreased, whereas the production of acetate was maximal. For a *k_L_a* value higher than 77 h^−1^, the biomass production was improved (Figure 1). A similar sequence in the organic acid production, according to the oxygen transfer conditions, was previously observed with other strains of *C. glutamicum*. Based on simulations using a genome‐scale metabolic model, Shinfuku et al. 23 highlighted the influence of oxygenation on the metabolism of *C. glutamicum* ATCC13032. Whereas for sufficient oxygen availability (aerobiosis), the glucose was converted to biomass and CO_2_, the decrease in oxygen transfer rate induced the acetate synthesis then, the lactate production and for the lowest oxygen transfer conditions the succinate synthesis 23. Using a microtiter plate‐based cultivation, Käß et al. 24 confirmed this impact of oxygenation on the metabolism of *C. glutamicum* ATCC13032 but also on the one of the l‐lysine producer *C. glutamicum* DM1933. With *C. glutamicum* ATCC13032, for maximum oxygen transfer rates, OTR_max_, from 5.7 to 3.27 mM h^−1^ (corresponding to *k_L_a* values between 25 and 14 h^−1^ considering an oxygen saturation of the medium at 0.232 mM), an increase in the organic acid production (lactate, succinate, and acetate) was observed 24. Concerning biomass synthesis, the growth of *C. glutamicum* DM1933 was highly limited for OTR_max_ lower than 14 mM h^−1^ (corresponding to *k_L_a* value of 60 h^−1^). Further increase in OTR_max_ had no beneficial effect on the growth of this strain. Similarly, with *C. glutamicum* 2262, the most significant effect of oxygenation level on the maximal specific growth (µ_max_) was noticed for *k_L_a* values inferior to 44 h^−1^ (Figure 2). The behavior of *C. glutamicum* 2262 was very close to the one of its related strain *C. glutamicum* 2262Δ*ldhA* designed for the succinate production. With this last strain, an organic acid production related to the oxygenation level was also observed. The maximal succinate and acetate production corresponded to *k_L_a* values between 10 and 19 h^−1^, and between 19 and 33 h^−1^, respectively, and a similar relationship between the *k_L_a* and the maximal biomass concentration was measured 18. Only a reduction in the conversion yields of glucose into organic acids was determined with *C. glutamicum* 2262. This could be explained by the lack of the metabolic pathway for lactate synthesis in *C. glutamicum* 2262Δ*ldhA* and, consequently, the need for this last strain to increase the succinate and acetate synthesis to maintain its intracellular redox balance.

It was reported that *C. glutamicum* was able to grow under anaerobic or deprived oxygen conditions, only by nitrate respiration 4, 6. However, it was recently demonstrated that in anaerobic conditions and even in the absence of nitrate, *C. glutamicum* can grow on glucose, fructose, sucrose, and ribose by mixed acid fermentation 9. In the present work, a residual growth was measured under highly limited oxygen conditions accompanied with organic acid production, during the cultures in shake flasks, performed at different *k_L_a* conditions. This could be attributed to the ability of *C. glutamicum* to simultaneously realize fermentation and aerobic respiration under micro‐aerobic conditions 25.

While glucose is considered as one of the preferential glucose for *C. glutamicum* growth, it was also reported that *C. glutamicum* was able to grow on a variety of other sugars or organic acids 3, 26, 27, 28 as a single or combined carbon and/or energy source. It was, however, reported that *C. glutamicum* ATCC 13032 was not able to grow on C4‐dicarboxylates as succinate, fumarate, and malate, as sole carbon source 29, 30. On the contrary, a weak growth of *C. glutamicum* R was observed during cultures on succinate as the sole carbon source 31. *Corynebacterium glutamicum* 2262 exhibited the same behavior as *C. glutamicum* ATCC13032 as no growth was noticed in the present study when succinate was the sole carbon molecule in the culture medium. Interestingly, a stop in the production and a re‐assimilation of the produced succinate, concomitant with a lower threshold glucose concentration of 9 g L^−1^ (50 mM; Figure 4), was observed in most of cultures performed in micro‐aerobic conditions, in shake flask. It was demonstrated that DccT, also called DcsT 31, and DctA were the only transporters for C4‐dicarboxylates in *C. glutamicum* ATCC 13032 29, 30. The expression of DccT encoding gene depended on the growth phase, being maximal in the exponential phase (early growth phase). Moreover, it was shown that an initial concentration of 9 g L^−1^ glucose exerted a repression on *dcsT* transcription 31. Similarly, repression of DctA homologs, identified in *E. coli* 32 and *Pseudomonas aeruginosa* 33, were measured for 7.2 and 9.9 g L^−1^ glucose, respectively. Thus, similar regulations of *dccT* and *dctA* in *C. glutamicum* 2262 could explain the re‐assimilation of succinate at a lower threshold of glucose concentration.

The influence of the transition phase from the aerobic to anaerobic phase on succinic acid production was previously investigated with genetically engineered strains of *C. glutamicum* 15 or *E. coli* 17. It was reported that the progressive deoxygenation based on a controlled dissolved oxygen concentrations (DOC) or gas flow rates, enhanced the succinate production by *C. glutamicum* 2262Δ*ldhA* 15. Martínez et al. 17 suggested that the presence of a micro‐aerobic phase at the end of the growth phase facilitated the bacterium to adjust its metabolic behavior by expressing genes necessary for the organic acid production during the anaerobic phase. Similarly, an upregulation of the gene encoding the glycolytic, fermentative enzymes, and enzymes of the reductive branch of the TCA cycle (*ldh*, *mdh*, and *pck*) was observed during a transition phase from aerobic to anaerobic conditions in *C. glutamicum* ATCC13032 25. Moreover, CO_2_ was described as a limiting substrate for the synthesis of succinate in several works 6, 12, 34, 35. In the present study, in order to evaluate the impact of process parameters control on the succinate production of *C. glutamicum* 2262, the optimized transition developed by Kaboré et al. 15 was applied during a fed‐batch culture with feedings of glucose combined with bicarbonate addition. Although the glucose residual concentration decreased under 9 g L^−1^ at 24 and 42 h of culture, no stop in the succinate production was measured. This difference compared to batch cultures in flask could be explained by the oxygenation level. Whereas the batch cultures were performed in micro‐aerobic condition, during the fed‐batch culture, succinate production occurred during an anaerobic phase. This suggests that succinate consumption could be influenced by the oxygenation of the culture medium. As substrate of succinate dehydrogenase, succinate is involved in the respiratory chain of *C. glutamicum* 36. This could explain the dependence of succinate consumption on oxygen availability. After the fifth pulse, the organic acid production reached a plateau despite the presence of residual glucose. This could be probably attributed to an inhibition by fermentation end‐products. Indeed, it was proved that, in *C. glutamicum*, glucose consumption and succinate production were significantly inhibited by the accumulation of extracellular succinate 37. Using this process, 93.6 g L^−1^ (793 mM) of succinate with a conversion yield of 0.91 mol mol^−1^ glucose (0.6 g g^−1^) were produced. The lactate final concentration (785 mM / 70.7 g L^−1^) was similar to the succinate final titer. Without genetic modification and by optimizing the process parameters, a wild‐type strain of *C. glutamicum* was then able to produce one of the highest concentration of succinate (93.57 g L^−1^) reported in the literature (Table 1) 38, 39. Indeed, several studies proved the ability of some prokaryotic and eukaryotic microbial strains (wild‐type or mutant strains) to overproduce this organic acid, from pure (glucose, glycerol, etc.) or complex carbon sources (wastes, hydrolysates, etc.) 40. The only non‐genetically modified bacterium producing a higher concentration of succinate than *C. glutamicum* 2262 was *Actinobacillus succinogenes* BE‐1, isolated from bovine rumen, with a final concentration of 145 g L^−1^ succinate. Such a concentration was reached using a fed‐batch culture coupled with an in situ product removal 41. To date, the highest concentration reported in the scientific literature was achieved with a genetically modified strain of *Yarrowia lipolytica PGC01003*, using optimized process conditions 41. This mutant strain overproduced succinate, using crude glycerol as carbon source. This yeast was able to produce 160 g L^−1^ succinate during a fed‐batch culture, with a productivity of 0.4 g L^−1^ h^−1^ 42. Considering *C. glutamicum*, the highest succinate concentration was also obtained from a genetically engineered strain, *C. glutamicum* R deleted for *ldhA* gene and in which pyruvate carboxylase activity was overexpressed 13. Using a fed‐batch process (feeding of glucose and carbonate), this strain produced 146 g L^−1^ succinate with a productivity of 2.48 g L^−1^ h^−1^, which was higher than the one reported for *Y. lipolytica PGC01003* 6, 7.

**Table 1:** Performances of succinate production processes using various microorganisms and substrates

Strains	Substrates	Process description	Concentration (g L^−1^)	Productivity (g L^−1^ h^−1^)	References
*C. glutamicum 2262*	Glucose	Fed‐Batch	93.6	1.42	This work
*C. glutamicum* BOL‐3/pAN6‐*gap*	Glucose	Fed‐Batch	146	2.48	14
*A. succinogenes BE‐1*	Glucose	Fed‐Batch	145	1.3	41
*A. succinogenes* 130z	Glucose	Batch	67.2	0.8	43
*A. succiniciproducens*	Glucose	Batch	50.3	2.1	44
*A. succiniciproducens*	Whey lactose	Fed‐Batch	34.7	1.02	45
*A. succinogenes 130Z*	Wheat hydrolysates	Batch	64.2	1.19	40
*Recombinant E.coli*	Food waste hydrolysate	Batch	29.9	0.48	46
*A. succinogenes*	Food waste hydrolysate	Batch	24.1	0.29	46
*C. glutamicum BL‐1/pVWEx1‐glpFKD*	Glycerol	Fed‐batch	9.3	0.42	47
*Y. lipolytica H222‐AZ2*	Glycerol	Fed‐batch	25	0.15	48
*Y. lipolytica PGC01003*	Crude glycerol	Fed‐Batch	160.2	0.4	42
*A. succinogenes*	Spent sulphite liquor	Batch	6.2	0.09	49

In this study, we demonstrated that the level of oxygenation and the availability of glucose were critical parameters for growth and organic acids production by *C. glutamicum*. A lower threshold glucose concentration, about 9 g L^−1^, was identified for the stop of succinate production. Then, based on the understanding of the physiological behavior of *C. glutamicum* 2262 in the context of a controlled microaerobiosis, an efficient process for the production of organic acids including succinic acid was proposed. This work demonstrated that, for some molecules of interest, naturally synthesized by the selected microbial catalyst, an accurate control of the process parameters could be as efficient as a genetic engineering methodology. Of course, some improvements of the proposed process are possible, for example, by combining the production on bioreactor with an online extraction of the end‐fermentation products.

## CONFLICT OF INTEREST

The authors have declared no conflict of interest.

## Supporting information


**Table S1** Composition of modified MCGC medium
**Table S2** Volumetric mass transfer coefficients (estimated by the correlation of Seletzky et al. 21) applied in the shake flask cultures performed during this study
