# Supplementary material for: Corynebacterium glutamicum, a natural overproducer of succinic acid?
Source: Eng Life Sci. 2020 Jan 20;20(5-6):205–15. doi: 10.1002/elsc.201900141 (PMC7447883; doi:10.1002/elsc.201900141)
Supplement: Supplementary file 1 — Table S1 Composition of modified MCGC medium Table S2 Volumetric mass transfer coefficients (estimated by the correlation of Seletzky et al. 21) applied in the shake flask cultures performed during this study [file ELSC-20-205-s001.pdf]

Table S1: Composition of modified MCGC medium

	Compounds	Final concentration		Unit
		Shake flask	Bioreactor	
Basis Salts	$Na_2HPO_4 12H_2O$	36.7	7.44	g L^{-1}
	KH_2PO_4	2.9	5.90	g L^{-1}
	NaCl	0.96	1.97	g L^{-1}
	$(NH_4)_2SO_4$	3.8	7.86	g L^{-1}
Salts A	$MgSO_4 7H_2O$	455.84	39.31	mg L^{-1}
	$FeSO_4 7H_2O$	45.6	3.93	mg L^{-1}
	$FeCl_3$	4.6	0.39	mg L^{-1}
Salts B	$ZnSO_4 7H_2O$	1.2	0.07	mg L^{-1}
	$CuCl_2 2H_2O$	0.46	0.03	mg L^{-1}
	$MnSO_4 H_2O$	4.6	0.26	mg L^{-1}
	$(NH_4)_6Mo_7O_{24} 4H_2O$	0.23	0.01	mg L^{-1}
	$Na_2B_4O_7 10H_2O$	0.46	0.03	mg L^{-1}
Vitamins	$CaCl_2$	94.9	8.18	mg L^{-1}
	Biotin	2.3	0.20	mg L^{-1}
	Thiamine	22.8	1.97	mg L^{-1}
	Deferoxamine	3.4	0.29	mg L^{-1}
	Betaïn	1937.32	0.20	mg L^{-1}
	Urea	3.9	-	g L^{-1}
	Glucose	33	85.5	g L^{-1}

Table S2: Volumetric mass transfer coefficients (estimated by the correlation of Seletzky et al. [21]) applied in the shake flask cultures performed during this study.

**Shaking frequency, *N* (rpm)**	**Flask volume, V_T (mL)**	**Filling volume, V_L (mL)**	**Flask diameter *d* (cm)**	**Shaking diameter, d_0 (cm)**	**Estimated k_La (h^{-1})**
0	250	100	8	0	0.6*
100	250	170	8	2.5	5
170	500	250	10	2.5	11
170	500	175	10	2.5	15
170	250	75	8	2.5	20
170	500	100	10	5	31
170	1000	100	12	5	44
170	1000	50	12	5	77
180	500	30	10	5	90
170	500	20	10	5	118

*: In the case of the culture without shaking, an experimental value of 0.6 h^{-1} was considered instead of the estimated value (0 h^{-1}).
